# Neoadjuvant Chemotherapy Followed by Surgery Versus Abdominal Radical Hysterectomy Alone for Oncological Outcomes of Stage IB3 Cervical Cancer—A Propensity Score Matching Analysis

**DOI:** 10.3389/fonc.2021.730753

**Published:** 2021-09-13

**Authors:** Weili Li, Wenling Zhang, Lixin Sun, Li Wang, Zhumei Cui, Hongwei Zhao, Danbo Wang, Yi Zhang, Jianxin Guo, Ying Yang, Wuliang Wang, Xiaonong Bin, Jinghe Lang, Ping Liu, Chunlin Chen

**Affiliations:** ^1^Department of Obstetrics and Gynecology, Nanfang Hospital, Southern Medical University, Guangzhou, China; ^2^Department of Gynecologic Oncology, Shanxi Provincal Cancer Hospital, Taiyuan, China; ^3^Department of Gynecologic Oncology, Affiliated Cancer Hospital of Zhengzhou University, Zhengzhou, China; ^4^Department of Obstetrics and Gynecology, The Affiliated Hospital of Qingdao University, Qingdao, China; ^5^Department of Obstetrics and Gynecology, Shengjing Hospital of China Medical University, Shenyang, China; ^6^Department of Gynecology, The First Hospital of China Medical University, Shenyang, China; ^7^Department of Obstetrics and Gynecology, Daping Hospital, Army Medical University, Chongqing, China; ^8^Department of Obstetrics and Gynecology, Xinqiao Hospital, Army Medical University, Chongqing, China; ^9^Department of Obstetrics and Gynecology, The Second Affiliated Hospital of He’nan Medical Unviersity, Zhengzhou, China; ^10^Department of Epidemiology, College of Public Health, Guangzhou Medical University, Guangzhou, China; ^11^Department of Obstetrics and Gynecology, Peking Union Medical College Hospital, Peking Union Medical College, Beijing, China

**Keywords:** cervical cancer, neoadjuvant chemotherapy, FIGO 2018, oncological outcomes, abdominal radical hysterectomy

## Abstract

**Objective:**

To compare the 5-year overall survival (OS) and disease-free survival (DFS) of patients with cervical cancer who received neoadjuvant chemotherapy followed by surgery (NACT) with those who received abdominal radical hysterectomy alone (ARH).

**Methods:**

We retrospectively compared the oncological outcomes of 1410 patients with stage IB3 cervical cancer who received NACT (n=583) or ARH (n=827). The patients in the NACT group were divided into an NACT-sensitive group and an NACT-insensitive group according to their response to chemotherapy.

**Results:**

The 5-year oncological outcomes were significantly better in the NACT group than in the ARH group (OS: 96.2% *vs.* 91.2%, respectively, *p*=0.002; DFS: 92.2% *vs.* 87.5%, respectively, *p*=0.016). Cox multivariate analysis suggested that NACT was independently associated with a better 5-year OS (HR=0.496; 95% CI, 0.281-0.875; *p*=0.015), but it was not an independent factor for 5-year DFS (HR=0.760; 95% CI, 0.505-1.145; *p*=0.189). After matching, the 5-year oncological outcomes of the NACT group were better than those of the ARH group. Cox multivariate analysis suggested that NACT was still an independent protective factor for 5-year OS (HR=0.503; 95% CI, 0.275-0.918; *p*=0.025). The proportion of patients in the NACT group who received postoperative radiotherapy was significantly lower than that in the ARH group (*p*<0.001). Compared to the ARH group, the NACT-sensitive group had similar results as the NACT group. The NACT-insensitive group and the ARH group had similar 5-year oncological outcomes and proportions of patients receiving postoperative radiotherapy.

**Conclusion:**

Among patients with stage IB3 cervical cancer, NACT improved 5-year OS and was associated with a reduction in the proportion of patients receiving postoperative radiotherapy. These findings suggest that patients with stage IB3 cervical cancer, especially those who are sensitive to chemotherapy, might consider NACT followed by surgery.

## Introduction

Globally, cervical cancer is a common malignant tumor in females, and it ranks fourth in terms of mortality (1, 2). In 2018, the International Federation of Gynecology and Obstetrics (FIGO) defined a new stage of cervical cancer, stage IB3, which is stage IB2 cervical cancer (FIGO 2009) but does not include cases of lymph node metastases (1, 3, 4). All cases involving pelvic lymph nodes and/or para-aortic lymph node metastases were classified as new stage IIIC. This classification raises questions as to whether the previous treatment recommendations for stage IB2 cervical cancer (FIGO 2009) are appropriate for the treatment of FIGO 2018 stage IB3. There is currently no clinical evidence supporting the choice of a specific treatment strategy in cases of FIGO 2018 stage IB3 cervical cancer.

It remains controversial whether NACT improves the oncological outcomes of patients with stage IB2 (FIGO 2009) cervical cancer compared to the standard treatment (5–12). Some studies have indicated that NACT improves patient prognosis (8, 10, 11), especially for patients who are sensitive to NACT (8, 11). Others have shown that the prognosis of cervical cancer patients who receive abdominal radical hysterectomy (ARH) or concurrent chemoradiotherapy is similar to that of patients who receive NACT followed by surgery. Some studies conducted in Japan and China have suggested that NACT does not improve the prognosis but reduces the proportion of patients receiving postoperative radiotherapy (12, 13).

The present study retrospectively analyzed the oncological outcomes of patients receiving NACT followed by surgery *versus* ARH alone based on the FIGO 2018 staging guidelines for stage IB3 cervical cancer. Based on the clinical diagnosis and treatment for cervical cancer in China (Four C) database, we aimed to assess the impact of NACT and its sensitivity on the oncological outcomes of stage IB3 cervical cancer patients.

## Methods

### Data Collection

This multicenter retrospective cohort study was approved by the Ethics Committee of the Southern Hospital of Southern Medical University (approval number NFEC-2017-135; clinical study registration number CHiCTR1800017778, International Clinical Trials Registry Platform Search Port, https://trialsearch.who.int/Default.aspx). The Four C database was created in cooperation with 47 hospitals in mainland China and includes data on 63,926 cervical cancer patients who were hospitalized between 2004 and 2018. The methods used for data entry, follow-up, double input and database establishment have been described previously (14, 15). The clinical staging of the patients in the database was revised according to the new FIGO 2018 staging system (1, 4).

### Inclusion and Exclusion Criteria

In this study, the inclusion criteria were as follows: age ≥ 18 years; histology indicating squamous cell carcinoma, adenocarcinoma or adenosquamous carcinoma; FIGO stage IB3 (FIGO 2018 staging system); NACT followed by surgery (NACT group) or ARH alone (ARH group); and QM type B or type C radical hysterectomy [Querleu and Morrow surgical classification system (16)] + pelvic lymphadenectomy ± para-aortic lymphadenectomy. Most of the patients in the NACT group received paclitaxel + platinum or platinum, generally in 1-2 courses. The NACT group received preoperative chemotherapy for 2-3 weeks followed by surgical treatment. The exclusion criteria were as follows: preoperative radiotherapy or radiotherapy and chemotherapy and the presence of other malignant tumors, pregnancy or stump cancer.

### Propensity Score Matching (PSM)

Because there may be differences in the clinical data (age, initial tumor diameter, and histological type) among the NACT group, ARH group and subgroups, we used the PSM method to balance those factors to ensure comparability.

Before and after PSM, Cox multivariate analysis was performed using the following factors: age, NACT, histology, surgical margin invasion, parametrial involvement, initial tumor diameter, deep stromal invasion, and lymphovascular space invasion (LVSI). The changes in tumor diameter before and after NACT were measured in accordance with the Response Evaluation Criteria in Solid Tumors (RECIST) standard (17). According to the RECIST standard, the numbers of patients with complete response, partial response, stable disease and progressive disease after NACT were 30 (7.09%), 284 (67.14%), 67 (15.84%) and 42 (9.93%), respectively. Patients exhibiting a complete response or a partial response in the NACT group were regarded as the NACT-sensitive group, while patients with stable disease or progressive disease were regarded as the NACT-insensitive group.

### Outcome Evaluation

The main observation outcomes were 5-year overall survival (OS) and 5-year disease-free survival (DFS) in the NACT group, the ARH group and the subgroups. OS was defined as the time from the date of diagnosis to death from any cause. DFS was defined as the time from the date of diagnosis to death or to the first evidence of recurrence. Patients with no evidence of recurrence or death were defined by the date of the last follow-up date or the last outpatient visit.

### Statistical Analysis

Statistical analyses were performed using SPSS 22.0 (SPSS, Inc., Chicago, IL, USA). Two independent sample *t* tests were used for continuous variables, and the *X^2^* test or the nonparametric test was used for categorical variables and graded variables. The log-rank test in the Kaplan-Meier (KM) method was used to compare the 5-year oncological outcomes (OS and DFS) of the two groups. The Cox proportional hazards regression model was used to calculate the hazard ratios (HRs) and 95% confidence intervals (CIs) for the multivariate analysis. In this study, logistic regression analysis was used. If the equal proportional hazard assumption was satisfied, logistic regression analysis was used to adjust the influence of other confounding factors on the pathological factors. If the equal proportion risk assumption was not satisfied, then nonequal proportion logistic regression was considered to analyze the influence of the research factors. Statistical significance was defined as *p*<0.05. Statisticians reviewed all statistical methods and statistical processes used in this study.

## Results

**Figure 1:**
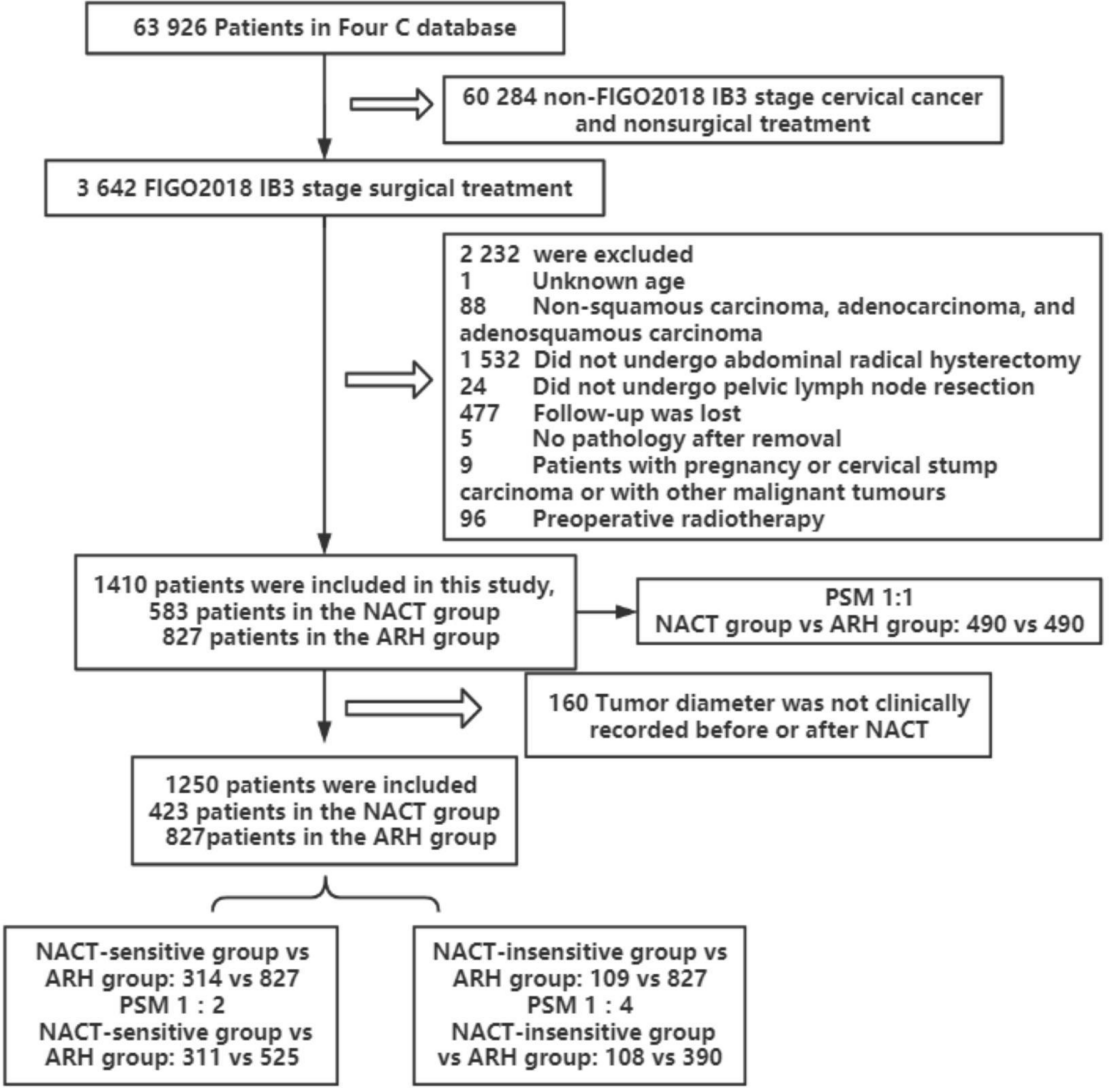
Flowchart of patients included in the analysis (NACT, neoadjuvant chemotherapy; ARH, abdominal radical hysterectomy).

A total of 1410 patients were included: 583 in the NACT group and 827 in the ARH group. The median follow-up time was 41 months. The median follow-up times for patients in the NACT and ARH groups were 40 months and 41 months, respectively. Information on tumor diameter before or after NACT was missing for 160 of the 583 patients in the NACT group, and information on tumor diameter both before and after NACT was available for 423 of the patients. Of those 423 patients, 352 had imaging data and 71 had no imaging data before NACT, while 225 had imaging data and 198 had no imaging data after NACT (**Table 1**). The data screening process is shown in **Figure 1**.

**Table 1:** Examination of patients with stage IB3 cervical cancer in the NACT group.

Examination	Before NACT (n = 423)	After NACT (n = 423)
B-ultrasound only	153 (36.2%)	166 (39.2%)
CT examination only	78 (18.4%)	24 (5.7%)
MRI examination only	60 (14.2%)	7 (1.7%)
B-ultrasound and CT examinations	39 (9.2%)	13 (3.1%)
B-ultrasound and MRI examinations	22 (5.2%)	14 (3.3%)
CT and MRI examinations	0	0
All three examinations	0	1 (0.2%)
Gynecological examination	423 (100%)	423 (100%)
Total	423 (100%)	423 (100%)

### Comparison of Oncological Outcomes Between the NACT Group and the ARH Group

Among the total study population, the difference in oncological outcomes between the NACT group (n=583) and the ARH group (n=827) was significant (OS: 96.2% *vs.* 91.2%, respectively, *p*=0.002; DFS: 92.2% *vs.* 87.5%, respectively, *p*=0.016). The 5-year oncological outcomes of the NACT group were better than those of the ARH group. Cox multivariate analysis suggested that NACT was independently associated with better 5-year OS (HR=0.496; 95% CI, 0.281-0.875; *p*=0.015) but was not an independent factor for 5-year DFS (HR=0.760; 95% CI, 0.505-1.145; *p*=0.189). LVSI positivity was an independent risk factor for 5-year DFS (HR=2.143; 95% CI, 1.343-3.419; *p*=0.001). The proportion of patients in the NACT group receiving postoperative radiotherapy was significantly lower than the proportion in the ARH group [220 (37.7%) *vs.* 439 (53.1%), respectively, *p<*0.001].

**Figure 2:**
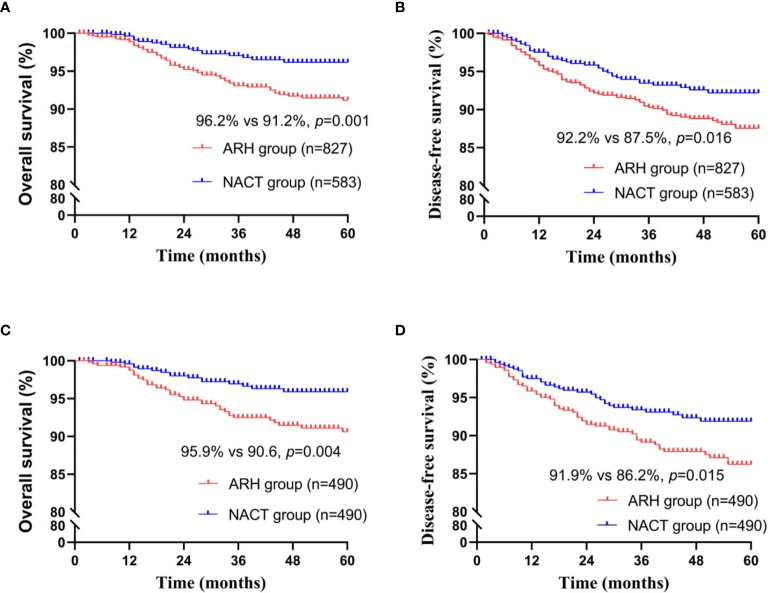
Survival curves of patients with stage IB3 cervical cancer in the NACT and ARH groups (unadjusted, panels **(A, B)**; adjusted, panels **(C, D)**; NACT, neoadjuvant chemotherapy; ARH, abdominal radical hysterectomy).

The NACT group and the ARH group were unbalanced at baseline. After 1:1 PSM, 490 cases were included in each group (**Table 2**, **Figure 2**). The 5-year oncological outcomes of the NACT group were better than those of the ARH group. Cox multivariate analysis indicated that NACT was still an independent protective factor for 5-year OS (HR=0.503; 95% CI, 0.275-0.918; *p*=0.025) but not an independent protective factor for 5-year DFS (*p*=0.114). LVSI positivity was found to be an independent risk factor for 5-year DFS (HR=2.082; 95% CI, 1.218-3.5557; *p*=0.007). The proportion of patients in the NACT group who received postoperative radiotherapy was significantly lower than that in the ARH group [189 (38.6%) *vs*. 250 (51.0%), respectively, *p<*0.001].

**Table 2:** Clinicopathologic characteristics of patients with stage IB3 cervical cancer in the NACT and ARH groups.

Variables	Unadjusted			Adjusted		
	NACT group (n = 583)	ARH group (n = 827)	*p*	NACT group (n = 490)	ARH group (n = 490)	*p*
**Age (years)**	45.38 ± 7.748	47.20 ± 8.672	<0.001	45.57 ± 7.336	45.66 ± 7.407	0.842
**Initial tumor diameter (cm)**	5.369 ± 0.871	5.387 ± 1.036	0.736	5.365 ± 0.872	5.376 ± 1.012	0.856
**Histological type**			0.208			0.942
SCC	514 (88.2%)	726 (87.8%)		434 (88.6%)	437 (89.2%)	
AC	59 (10.1%)	75 (9.1%)		48 (9.8%)	46 (9.4%)	
SAC	10 (1.7%)	26 (3.1%)		8 (1.6%)	7 (1.4%)	
**Parametrial involvement**			0.358			0.762
Negative	576 (98.8%)	821 (99.3%)		485 (99.0%)	484 (98.8%)	
Positive	7 (1.2%)	6 (0.7%)		5 (1.0%)	6 (1.2%)	
**Vaginal margin invasion**			0.844			1.000
Negative	576 (98.8%)	818 (98.9%)		485 (99.0%)	485 (99.0%)	
Positive	7 (1.2%)	9 (1.1%)		5 (1.0%)	5 (1.0%)	
**LVSI**			<0.001			<0.001
Negative	549 (94.2%)	724 (87.5%)		463 (94.5%)	421 (85.9%)	
Positive	34 (5.8%)	103 (12.5%)		27 (5.5%)	69 (14.1%)	
**Stromal invasion**			<0.001			<0.001
≤1/2	273 (46.8%)	237 (28.6%)		227 (46.3%)	138 (28.2%)	
>1/2	256 (43.9%)	549 (66.4%)		220 (44.9%)	330 (67.3%)	
Unknown	54 (9.3%)	41 (5.0%)		43 (8.8%)	22 (4.5%)	
**Postoperative radiotherapy**			<0.001			<0.001
No	363 (62.3%)	388 (46.9%)		301 (61.4%)	240 (49.0%)	
Yes	220 (37.7%)	439 (53.1%)		189 (38.6%)	250 (51.0%)	

NACT, neoadjuvant chemotherapy; ARH, abdominal radical hysterectomy; SCC, squamous cell carcinoma; AC, adenocarcinoma; SAC, adenosquamous carcinoma; LVSI, lymphovascular space invasion.

The NACT group had a significantly lower rate of detection of postoperative LVSI and deep stromal invasion than in the ARH group [before matching: LVSI, 34 (5.8%) *vs.* 103 (12.5%), respectively, *p*<0.001, deep stromal invasion, 256 (43.9%) *vs.* 549 (66.4%), respectively, *p*<0.001; after matching: LVSI, 27 (5.5%) *vs.* 69 (14.1%), respectively, *p*<0.001, deep stromal invasion: 220 (44.9%) *vs.* 330 (67.3%), respectively, *p*<0.001]. There was no statistically significant difference in parauterine infiltration or vaginal margin infiltration between the two groups. Logistic regression analysis showed that NACT was an independent protective factor for postoperative cervical deep stromal invasion and LVSI (*p*<0.001).

### Comparison of Oncological Outcomes Between the NACT-Sensitive Group and the ARH Group

The NACT-sensitive group consisted of 314 patients. Among the total study population, the 5-year oncological outcomes of the NACT-sensitive group with stage IB3 cervical cancer were significantly better than those of the ARH group (OS: 97.1% *vs.* 91.2%, respectively, *p*=0.002; DFS: 92.0% *vs.* 87.5%, respectively, *p*=0.032). Cox multivariate analysis suggested that NACT sensitivity was an independent protective factor for 5-year OS (HR=0.354; 95% CI, 0.159-0.788; *p*=0.011) but not an independent protective factor for 5-year DFS. LVSI positivity was found to be an independent risk factor for 5-year DFS. The proportion of patients receiving postoperative radiotherapy in the NACT-sensitive group was significantly lower than that in the ARH group [108 (34.4%) *vs.* 439 (53.1%), respectively, *p<*0.001].

**Figure 3:**
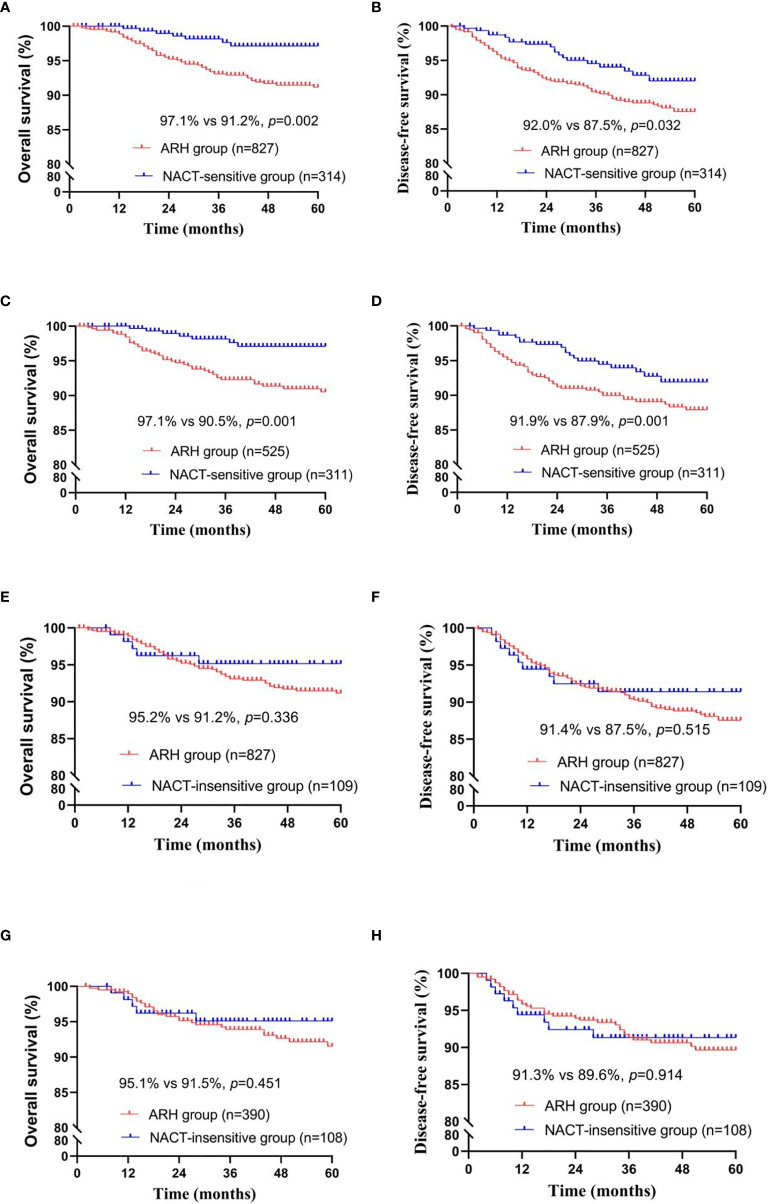
Survival curves of patients with stage IB3 cervical cancer in the NACT-sensitive/NACT-insensitive and ARH groups [NACT-sensitive: unadjusted, panels **(A, B)**; adjusted, panels **(C, D)**; NACT-insensitive: unadjusted, panels **(E, F)**; adjusted, panels **(G, H)**; NACT, neoadjuvant chemotherapy; ARH, abdominal radical hysterectomy].

After 1:2 PSM, 311 patients and 525 patients were included in the NACT-sensitive group and the ARH group, respectively (**Table 3**, **Figure 3**). The 5-year oncological outcomes of the NACT sensitivity group were better than those of the ARH group (OS: 97.1% *vs.* 90.5%, respectively, *p*=0.001; DFS: 91.9% *vs.* 87.9%, respectively, *p*=0.041). Cox multivariate analysis suggested that NACT sensitivity was an independent protective factor for 5-year OS (HR=0.326; 95% CI, 0.144-0.741; *p*=0.007) but not an independent protective factor for 5-year DFS. LVSI positivity was an independent risk factor for 5-year DFS. The proportion of patients receiving postoperative radiotherapy in the NACT-sensitive group was significantly lower than that in the ARH group [108 (34.7%) *vs.* 272 (51.8%), respectively, *p<*0.001].

**Table 3:** Clinicopathologic characteristics of patients with stage IB3 cervical cancer in the NACT-sensitive and ARH groups.

Variables	Unadjusted			Adjusted		
	NACT-sensitive group (n = 314)	ARH group (n = 827)	*p*	NACT-sensitive group (n = 311)	ARH group (n = 525)	*p*
**Age (years)**	45.13 ± 7.672	47.20 ± 8.672	<0.001	45.07 ± 7.300	45.97 ± 7.154	0.080
**Initial tumor diameter(cm)**	5.401 ± 0.817	5.387 ± 1.036	0.815	5.400 ± 0.820	5.414 ± 1.084	0.834
**Histological type**			0.377			0.912
SCC	284 (90.5%)	726 (87.8%)		281 (90.4%)	470 (89.5%)	
AC	24 (7.6%)	75 (9.1%)		24 (7.7%)	43 (8.2%)	
SAC	6 (1.9%)	26 (3.1%)		6 (1.9%)	12 (2.3%)	
**Parametrial involvement**			0.595			0.916
Negative	310 (98.7%)	821 (99.3%)		307 (98.7%)	520 (99.0%)	
Positive	4 (1.3%)	6 (0.7%)		4 (1.3%)	5 (1.0%)	
**Vaginal margin invasion**			1.000			1.000
Negative	311 (99.0%)	818 (98.9%)		308 (99.0%)	519 (98.9%)	
Positive	3 (1.0%)	9 (1.1%)		3 (1.0%)	6 (1.1%)	
**LVSI**			0.001			<0.001
Negative	297 (94.6%)	724 (87.5%)		294 (94.5%)	456 (86.9%)	
Positive	17 (5.4%)	103 (12.5%)		17 (5.5%)	69 (13.1%)	
**Stromal invasion**			<0.001			<0.001
≤1/2	164 (52.2%)	237 (28.6%)		163 (52.5%)	148 (28.2%)	
>1/2	119 (37.9%)	549 (66.4%)		118 (37.9%)	355 (67.6%)	
Unknown	31 (9.9%)	41 (5.0%)		30 (9.6%)	22 (4.2%)	
**Postoperative radiotherapy**			<0.001			<0.001
No	206 (65.6%)	388 (46.9%)		203 (65.3%)	253 (48.2%)	
Yes	108 (34.4%)	439 (53.1%)		108 (34.7%)	272 (51.8%)	

NACT, neoadjuvant chemotherapy; ARH, abdominal radical hysterectomy; SCC, squamous cell carcinoma; AC, adenocarcinoma; SAC, adenosquamous carcinoma; LVSI, lymphovascular space invasion.

The NACT-sensitive group had a significantly lower rate of detection of postoperative LVSI and deep stromal invasion than the ARH group [before matching: LVSI, 17 (5.4%) *vs.* 103 (12.5%), respectively, *p*<0.001, deep stromal invasion, 119 (37.9%) *vs.* 549 (66.4%), respectively, *p*<0.001; after matching: LVSI, 17 (5.5%) *vs.* 69 (13.1%), respectively, *p*<0.001, deep stromal invasion, 118 (37.9%) *vs.* 355 (67.6%), respectively, *p*<0.001). There was no statistically significant difference in surgical margin invasion or parametrial involvement between the two groups. Logistic regression analysis showed that NACT sensitivity was an independent protective factor for postoperative cervical deep stromal invasion and LVSI (*p*<0.05).

### Comparison of Oncological Outcomes Between the NACT-Insensitive Group and the ARH Group

There were 109 patients in the NACT-insensitive group. There was no statistically significant difference in the 5-year oncological outcomes between the NACT-insensitive group with stage IB3 cervical cancer and the ARH group. Cox multivariate analysis indicated that NACT insensitivity was not an independent factor for 5-year oncological outcomes. The proportion of patients receiving postoperative radiotherapy in the NACT-insensitive group was similar to that in the ARH group, and the difference was not significant (*p*=0.606).

After 1:4 PSM, the NACT-insensitive group and the ARH group included 108 cases and 390 cases, respectively (**Table 4**, **Figure 3**). There were no significant differences in the 5-year oncological outcomes or in the proportion of patients receiving radiotherapy between the NACT-insensitive group and the ARH group (*p*=0.369).

**Table 4:** Clinicopathologic characteristics of patients with stage IB3 cervical cancer in the NACT-insensitive and ARH groups.

Variables	Unadjusted			Adjusted		
	NACT-insensitive group (n = 109)	ARH group (n = 827)	*p*	NACT-insensitive group (n = 108)	ARH group (n = 390)	*p*
**Age (years)**	44.98 ± 7.93	47.20 ± 8.672	0.011	44.19 ± 7.721	46.01 ± 6.905	0.283
**Initial tumor diameter (cm)**	5.363 ± 1.002	5.387 ± 1.036	0.816	5.357 ± 1.005	5.316 ± 0.892	0.679
**Histological type**			0.26			0.74
SCC	100 (91.8%)	726 (87.8%)		99 (91.7%)	350 (89.7%)	
AC	8 (7.3%)	75 (9.1%)		8 (7.4%)	33 (8.5%)	
SAC	1 (0.9%)	26 (3.1%)		1 (0.9%)	7 (1.8%)	
**Parametrial involvement**			1.000			1.000
Negative	108 (99.1%)	821 (99.3%)		107 (99.1%)	385 (98.7%)	
Positive	1 (0.9%)	6 (0.7%)		1 (0.9%)	5 (1.3%)	
**Vaginal margin invasion**			0.836			1.000
Negative	107 (98.2%)	818 (98.9%)		106 (98.1%)	385 (98.7%)	
Positive	2 (1.8%)	9 (1.1%)		2 (1.9%)	5 (1.3%)	
**LVSI**			0.016			0.016
Negative	104 (95.4%)	724 (87.5%)		103 (95.4%)	340 (87.2%)	
Positive	5 (4.6%)	103 (12.5%)		5 (4.6%)	50 (12.8%)	
**Stromal invasion**			0.815			0.491
≤1/2	34 (31.2%)	237 (28.6%)		34 (31.5%)	104 (26.6%)	
>1/2	69 (63.3%)	549 (66.4%)		68 (63.0%)	269 (69.0%)	
Unknown	6 (5.5%)	41 (5.0%)		6 (5.6%)	17 (4.4%)	
**Postoperative radiotherapy**			0.606			0.369
No	54 (49.5%)	388 (46.9%)		54 (50.0%)	176 (45.1%)	
Yes	55 (50.5%)	439 (53.1%)		54 (50.0%)	214 (54.9%)	

NACT, neoadjuvant chemotherapy; ARH, abdominal radical hysterectomy; SCC, squamous cell carcinoma; AC, adenocarcinoma; SAC, adenosquamous carcinoma; LVSI, lymphovascular space invasion.

The NACT-insensitive group had a significantly lower rate of detection of postoperative LVSI than the ARH group [before matching: LVSI, 5 (4.6%) *vs.* 103 (12.5%), respectively, *p*<0.001; after matching: LVSI, 5(4.6%) *vs.* 50(12.8%), respectively, *p*<0.001]. There was no statistically significant difference in deep stromal infiltration, surgical margin invasion or parametrial involvement between the two groups. Logistic regression analysis showed that NACT, even in the insensitive group, was an independent protective factor for postoperative LVSI (*p*<0.05).

## Discussion

The present study found that NACT improved the 5-year OS of patients with FIGO 2018 stage IB3 cervical cancer and reduced the positive rates of postoperative LVSI and cervical deep stromal infiltration, thereby reducing the proportion of patients receiving postoperative radiotherapy. These results suggest that patients with stage IB3 cervical cancer undergoing ARH, especially NACT-sensitive patients, might receive NACT to help reduce the postoperative complications caused by radiotherapy.

The previous literature has mainly focused on the effect of NACT on the prognosis of stage IB2 or locally advanced cervical cancer (FIGO 2009). Some studies have considered that surgery after NACT improves patient prognosis (8–11, 18–20), especially for patients who are sensitive to NACT (8, 11, 19, 20). Sardi et al. (10) found in a prospective randomized controlled study of stage IB cervical squamous cell carcinoma (d>4cm) that the survival and DFS of patients who received NACT were better than those of patients who underwent direct surgery or postoperative radiotherapy; this finding is consistent with the conclusion of the present study. Cai et al. (8) also obtained similar results for patients with stage IB (d>4 cm) cervical carcinoma. Chen et al. (11) included patients with stage IB2-IIB cervical cancer and found that modified preoperative NACT was beneficial in reducing tumor size, eliminating pathological risk factors, and improving the prognosis of responders. Gadducci et al. (20) found that NACT followed by surgery is an effective treatment for stage IB2-IIB cervical cancer and that the pathological response to NACT is the most important factor affecting the prognosis.

Studies have also found that the prognosis of patients who receive NACT followed by surgery is similar to that of patients who receive ARH alone or concurrent radiotherapy (12, 13, 21, 22). Some studies (12, 13) have suggested that although NACT does not improve prognosis, it reduces the proportion of patients who require postoperative radiotherapy. Katsumata et al. (12) showed that NACT followed by surgery does not improve OS compared to direct surgery in patients with stage IB2, IIA2 and IIB cervical cancer but that it may reduce the proportion of patients receiving postoperative radiotherapy. These differing results from those obtained in our study may be due to later staging (more than half were stage IIB) and the inclusion of patients with lymph node metastases. Yang et al. (13) reported no difference in 1-year and 3-year OS or DFS between the NACT group and the direct operation group in patients with stage IB2, IIA2 and IIB cervical cancer, and the authors reported that the number of patients receiving postoperative radiotherapy was reduced in the NACT group. That study included many patients with late-stage disease as more than half of the included patients were stage IIB, and patients with stage IB2 accounted for approximately 17.4% (19/109) and 22.7% (25/110) of the patients in the NACT group and the direct operation group, respectively, which may explain why their results differ from those obtained in our study. Most of the cervical cancer patients included in the studies by Duenas-Gonzalez et al. (9) and Gupta et al. (21), who analyzed the prognosis between chemoradiotherapy and NACT followed by surgery, were at or above stage IIB.

Due to the controversy over the efficacy of NACT, the international community has generally maintained a cautious attitude. The 2021 National Comprehensive Cancer Network (NCCN) guidelines (23) recommend concurrent chemoradiotherapy for patients with stage IB3 cervical cancer (level of evidence 1) or radical hysterectomy + PL ± PAL (level of evidence 2B). The FIGO Cancer Report 2018 (1) also emphasizes that NACT for cervical cancer is only recommended for use in areas that lack radiotherapy facilities, in prospective studies and in clinical trials. For patients with stage IB2 cervical cancer (FIGO 2009), NACT followed by surgery or ARH is also common in some institutions in Asia and Europe (11, 12, 20). Therefore, there is no multicenter or large-sample clinical evidence that addresses whether the recommended treatment for stage IB2 cervical cancer based on the FIGO stage as defined in 2009 is still suitable for the treatment of stage IB3 defined according to the FIGO 2018 guidelines.

Compared to radiotherapy, surgery has the following two recognized advantages: (1) it preserves ovarian function and (2) it causes less damage in terms of vaginal shortening and sexual function, especially in young women. For these reasons, more patients in China with stage IB3 cervical cancer choose surgery. Previous studies (9, 11–13, 20, 21) mostly evaluated patients with cervical cancer stage IB2 and IIA2 (FIGO 2009), or even included those with more advanced cervical cancer, with a low proportion of patients with stage IB2 cervical cancer (FIGO 2009). Moreover, the FIGO 2018 guidelines for stage IB3 cervical cancer excluded lymph node metastasis, and stage IB3 is an earlier stage than stages IIA2 and IIB. To date, there have been no studies discussing the therapeutic effect of NACT followed by surgery compared to ARH on stage IB3 cervical cancer. Based on the Four C database, it was concluded that the 5-year OS of the NACT group was better than that of the ARH group in patients with stage IB3 cervical cancer, especially for patients with NACT sensitivity. Thus, compared to ARH alone, NACT followed by surgery may be a better treatment option.

In the present study, there were 423 patients for whom tumor diameter records before and after NACT treatment were available. Of these, 314 were NACT-sensitive patients with an effective rate of 74.23%, and 109 were NACT-insensitive patients with an inefficiency of 25.77%. The effective rate in this study was slightly lower than that reported in the literature, which ranges from 79.5% to 84.6% (8, 24); this result may be related to the elimination of 160 patients with unrecorded tumor diameters before or after NACT from the study group, resulting in a certain degree of bias. The oncological outcomes and the radiotherapy rate of NACT-insensitive patients were comparable to those of ARH patients, and NACT insensitivity did not improve patient prognosis but rather increased hospitalization costs and chemotherapy side effects. Therefore, these findings suggest that screening for NACT-sensitive patients is of great significance (11, 20). Sun et al. found that radiomic analysis effectively screens for NACT-sensitive patients (25).

The FIGO Cancer Report 2018 mentions that NACT may conceal some pathological results, that it may affect decisions regarding whether adjuvant treatment should be administered after surgery and that patients with large tumor diameters and pathological types of adenocarcinoma have a low response rate to NACT (1). Some studies have noted that the prognosis of patient who receive surgery after NACT may be related to the patient’s age, initial tumor diameter and pathological type (26). The present study used age, initial tumor diameter, and pathological type as the baseline, and there were no significant differences in these parameters between the two groups. Therefore, we concluded that NACT or NACT sensitivity improves the OS of patients and reduces the proportion of patients requiring postoperative radiotherapy, perhaps because NACT or NACT sensitivity reduces the intermediate-risk factors for postoperative pathology. Li et al. retrospectively found that NACT reduces the positive rate of “intermediate-risk factors” for LVSI and deep stromal invasion in patients with cervical squamous cell carcinoma stage IB2 and IIA2 as defined by FIGO (2009) (27). Our study suggested that NACT and NACT-sensitive patients with stage IB3 cervical cancer had reduced positivity for LVSI and deep stromal infiltration. According to the “Sedlis criteria” for intermediate-risk factors based on the NCCN guidelines (23, 28), these patients had a reduced need for postoperative radiotherapy. In univariate analysis, the 5-year DFS of both the NACT group and the NACT-sensitive group was superior to that of the ARH group. Cox multivariate analysis showed that NACT or NACT sensitivity was not an independent protective factor for DFS. There may be a certain interaction between NACT and LVSI. The DFS of the NACT group was higher than that of the ARH group because the postoperative pathology was not matched in the univariate analysis. However, the interaction between the NACT and LVSI was considered in the Cox multivariate analysis, and LVSI positivity was an independent risk factor for DFS. NACT and NACT sensitivity are not independent protective factors for DFS.

Our study is one of the first population-based studies to compare 5-year OS and DFS after treatment with NACT followed by surgery and after treatment with ARH alone in stage IB3 cervical cancer patients and related subgroups. First, the strength of the present study was its large sample size. Our study analyzed a large cohort of cervical cancer patients who were treated over a 14-year period at 47 hospitals. Second, this study may be the first to investigate the oncological outcomes of patients with stage IB3 cervical cancer (as defined by FIGO 2018) who were treated with NACT followed by surgery or with ARH alone. In previous studies, most patients with stage IB2 and IIA2 or more advanced stage cervical cancer (FIGO 2009) were combined and discussed. The proportion of patients with stage IB2 cervical cancer in those studies was low, and patients with lymph node metastasis were included. In addition, patients who underwent laparoscopic surgery were not excluded from the surgical approach. Because the 2018 Laparoscopic Approach to Cervical Cancer (LACC) Trial (29) indicated that laparoscopic surgery is not conducive to the oncological outcomes of patients with cervical cancer, patients who received laparoscopic surgery were excluded from our study. Ferrandina et al. found that minimally invasive radical surgery and open radical surgery were associated with similar rates of recurrence and death in patients with locally advanced cervical cancer whose cancers were managed by surgery after chemoradiation (30). This finding is worthy of further discussion regarding the oncological outcomes of laparoscopic and minimally invasive surgery in patients with stage IB3 cervical cancer in the NACT group. The considerations described above may account for the differences in the conclusions between previous studies and the present study.

The present study has several limitations. First, as a retrospective study, it does not provide the highest level of evidence. Second, the NACT treatment plan may have had an impact on prognosis. The present study did not detail the impact of different plans and different treatment courses on patient prognosis. The present study analyzed only the proportion of patients who received postoperative radiotherapy and did not analyze postoperative complications. Third, all patients with stage IB3 cervical cancer in this study received surgical treatment, although in other countries most of these patients are treated with concurrent chemoradiotherapy; this difference may make the conclusions not broadly applicable. However, the latest randomized controlled study (EORTC 55994) confirmed that the efficacy of NACT followed by surgery is similar to that of concurrent chemoradiotherapy in patients with stage IB2-IIB cervical cancer (FIGO 2009) with no difference in 5-year OS (72% and 76%, respectively, *p*=0.332) (31). Nama et al. (32) also reported that there is no single randomized controlled trial in which direct surgery for stage IB2 (FIGO 2009) is compared with chemoradiotherapy; thus, this area requires further research. The current results suggest that surgical treatment is an option for stage IB3 cervical cancer. In addition, our analysis of NACT sensitivity excluded patients whose tumor diameters before or after NACT were unknown. The clinical records were not standardized, which may have caused selection bias. Finally, the identification of NACT-sensitive patients before the administration of NACT was also an important prognostic factor.

## Conclusion

In conclusion, the results of the present study suggest that in patients with stage IB3 cervical cancer as defined in FIGO 2018, NACT before ARH can increase 5-year OS, reduce intermediate-risk factors for postoperative pathology, and reduce the proportion of patients requiring postoperative radiotherapy; the latter may reduce the complications caused by radiotherapy. Therefore, patients with stage IB3 cervical cancer, especially those who exhibit NACT sensitivity, might opt for receive NACT before ARH.

## Data Availability Statement

The original contributions presented in the study are included in the article/supplementary material. Further inquiries can be directed to the corresponding authors.

## Author Contributions

WL, WZ, LS, and LW contribute equally to the work. WL: literature search, data collection, data analysis and interpretation, Methodology. WZ: Data Curation, Writing-Original Draft, Writing-Review & Editing. LS: Investigation, Writing-Original Draft, Writing-Review & Editing. LW: literature search, data collection, Investigation, Writing-Review & Editing. ZC: Investigation, Resources. HZ: Investigation, Resources. DW: Investigation, Resources. YZ: Investigation, Resources. JG: Investigation, Resources. YY: Investigation, Resources. WW: Investigation, Resources. XB: Formal analysis. JL: Supervision, Conceptualization. PL: Supervision, Conceptualization, Project administration. CC: Supervision, Conceptualization, Project administration, Funding acquisition. All authors contributed to the article and approved the submitted version.

## Funding

This study was supported by the National Science and Technology Support Program 252 of China (2014BAI05B03); the National Natural Science Fund of Guangdong 253 (2015A030311024) and the Science and Technology Plan of Guangzhou (158100075).

## Conflict of Interest

The authors declare that the research was conducted in the absence of any commercial or financial relationships that could be construed as a potential conflict of interest.

## Publisher’s Note

All claims expressed in this article are solely those of the authors and do not necessarily represent those of their affiliated organizations, or those of the publisher, the editors and the reviewers. Any product that may be evaluated in this article, or claim that may be made by its manufacturer, is not guaranteed or endorsed by the publisher.
